# Ras and autophagy in cancer development and therapy

**DOI:** 10.18632/oncotarget.1775

**Published:** 2014-02-13

**Authors:** Eran Schmukler, Yoel Kloog, Ronit Pinkas-Kramarski

**Affiliations:** ^1^ Department of Neurobiology. Tel-Aviv University, Ramat-Aviv, Israel

**Keywords:** Autophagy, Ras, cancer, oncogene

## Abstract

Autophagy, a process of self-degradation and turnover of cellular components, plays a complex role in cancer. Evidence exists to show that autophagy may support tumor growth and cell survival, whereas it can also contribute to tumor suppression and have anti-survival characteristics in different cellular systems. Numerous studies have described the effects of various oncogenes and tumor suppressors on autophagy. The small GTPase Ras is an oncogene involved in the regulation of various cell-signaling pathways, and is mutated in 33% of human cancers. In the present review, we discuss the interplay between Ras and autophagy in relation to oncogenesis. It appears that Ras can upregulate or downregulate autophagy through several signaling pathways. In turn, autophagy can affect the tumorigenicity driven by Ras, resulting in either tumor progression or repression, depending on the cellular context. Furthermore, Ras inhibitors were shown to induce autophagy in several cancer cell lines.

## INTRODUCTION

Autophagy is a highly conserved cellular degradation mechanism. Autophagy is active at basal levels as part of the cell homeostasis machinery, but it can be also upregulated under stress conditions, such as nutrients deprivation or certain growth factors withdrawal. During autophagy, a portion of the cytoplasm can be degraded (non-selective autophagy); however, several types of selective autophagy also exist, such as degradation of mitochondria (mitophagy). Induction of autophagy usually promotes cell survival; nonetheless, it can also lead to cell death, known as type II PCD or autophagic cell death [1].

Autophagy was found to be involved in various pathologies, including cancer formation and progression [2]. It can play both anti- and pro-tumorigenic roles, depending on tumor type, stage and cellular context. The tumor-suppressive properties of autophagy mostly relate to the elimination of genotoxic stress (e.g. reactive oxygen species), thus protecting DNA integrity. Under stress conditions such as those inducing DNA damage or anti-cancer therapy, cells can undergo autophagic cell death [2, 3]. On the other hand, its tumor promoting features are mainly attributed to its pro-survival roles under stress conditions, such as decreased blood supply, metabolic stress, protein aggregation and loss of extracellular matrix (ECM) [2, 4], which are usually imposed on cancer cells. In view of that, many anti-cancer drugs were shown to induce protective autophagy, which allows cancer cells to overcome the treatment [3].

Ras is a membrane-anchored protein, which participates in generation of multiple signaling cascades within the cell. Signaling pathways induced by Ras may promote cell tumorigenicity, which makes Ras a major target for anti-cancer intervention [5]. Although Ras inhibitors are used experimentally, currently there are no approved anti-Ras drugs [5].

The crosstalk between Ras and autophagy has been studied extensively. Ras can modulate the levels of autophagy in cancer cells, while autophagy may affect the progression of Ras-driven tumors. In the present review, we will discuss the link between Ras signaling and autophagy in the context of cancer, and the effect of anti-Ras agents on this process.

### Major proteins involved in autophagy regulation

Autophagy is a multi-step process that involves numerous proteins encoded by autophagy-related genes (*ATG*s). Several regulatory proteins control the induction step of autophagy. Autophagic stimuli (e.g. nutrients deprivation) usually lead to inhibition of the class I PI3K/Akt/mTOR1 pathway [6]. In mammals, phosphorylation of ULK1 (Atg1) and Atg13 by mTOR1 inhibits the ULK1/Atg13/FIP200 complex, which is essential for the induction of autophagy, thus, making mTOR1 a major repressor of autophagy.

Autophagy induction also requires the activation of class III PI3K complex which includes among others, the Vps34 and beclin 1/Atg6 proteins [7]. Activation of this complex is mainly regulated through the binding of beclin 1 to Vps34, while the later acts as the catalytic subunit. This interaction is promoted through several regulatory proteins (e.g. UVRAG and Bif-1). Conversely, anti-apoptotic Bcl-2 proteins (e.g. Bcl-2, Bcl-x_L_ and Mcl-1) interact with beclin 1 and interfere with beclin 1/Vps34 association [7, 8]. Under pro-autophagic conditions, beclin 1 is released from Bcl-2 proteins, allowing the induction of autophagy. The targeting and inhibition of PI3Ks may be used for the suppression of autophagy, e.g. by the use of 3-methyladenine (3-MA), which affects Vps34 [6].

Induction of autophagy leads to the formation of the phagophore, a double membrane structure, which engulfs the cargo destined for degradation. The phagophore then elongates to form a mature vesicle, known as the autophagosome, which sequesters the cargo. Autophagosome formation is mediated by two ubiquitin-like systems consisting of several proteins, including Atg5, Atg7, Atg12 and Atg8/LC3-I [9]. The products of these systems are Atg5/Atg12/Atg16 tetramers and Atg8-phosphatidylethanolamine (PE) conjugate, also designated LC3-II.

The final step of autophagy involves the fusion of the autophagosome with the lysosome, degradation of the cargo by the lysosomal hydrolases and recycling of the building blocks back to the cytosol. Blockage of autophagy using agents perturbing lysosome acidification (e.g. chloroquine) or protease inhibitors (e.g. pepstatin A), leads to the accumulation of autophagosomes in the cell and might be of toxic nature [10].

Aside from the Atg's and the mTOR1 pathway, other proteins also participate in the process of autophagy. For example, p62/SQSTM1 was shown to mediate the degradation of ubiquitinated protein aggregates and mitochondria [11, 12]. Some of Ras downstream substrates, such as JNK and ERK were also shown to regulate autophagy (will be discussed below).

### Autophagy in cancer

Mutations and deletions of autophagy related genes, as well as aberrant expression of autophagy-mediating proteins, have been linked to several cancers in humans, including the loss of *BECN1* (coding for beclin 1) in breast, ovarian and prostate cancer [13, 14], mutations in *UVRAG*, *ATG12* and *ATG5* in colon and gastric cancer [15, 16], overexpression of p62/SQSTM1 in breast and lung cancer [17] and low expression levels of Bif-1 in prostate cancer [18].

Many human oncogenes or tumor suppressor genes and their protein products were shown to affect autophagy, and vice-versa. These include Ras (which will be discussed below), EGFR and HER2/Neu [19], p53 [20], BCR-Abl [21, 22], PTEN [23, 24], Myc [25, 26], NF-κB [27] and estrogen receptor [28]. Consequently, anti-cancer drugs targeting these proteins were also shown to modulate autophagy [20, 29].

### Ras signaling and autophagy regulation

Ras family of small GTPases consists of four highly related members, K-Ras (4A and 4B), H-Ras and N-Ras [30]. These proteins are normally located at the inner leaflet of the plasma membrane, where they take part in the transmission of signals through interaction with multiple effectors. Activation of Ras is initiated by cell surface receptors, which can induce RasGEFs (guanine-nucleotide exchange factors, e.g. SOS) to exchange GDP with GTP on Ras. Once activated, Ras stimulates diverse downstream effectors leading to the initiation of an array of cellular signaling networks, including: class I PI3K/Akt/mTOR1, Raf-1/MEK/ERK, RalGDS, PLCε/PKC and Rac1/JNK pathways [30]. RasGAPs (GTPases-activating proteins, e.g. neurofibromin) facilitate Ras inactivation by enabling its GTPase activity, which leads to the hydrolysis of GTP [30].

Mutational activation of *RAS* genes is involved in 33% of human cancers, with mutation of *KRAS* being the most prevalent (21.6% of human cancers) [31]. *KRAS* mutations are particularly associated with the most lethal malignancies: lung, colon and pancreatic cancer [32]. While wild-type Ras cycles between the active and inactive states, the oncogenic mutant Ras binds GTP in an unregulated manner, and is, therefore, constitutively active. This aberrant activity stems from a point mutation at residues 12, 13 or 61, which renders Ras proteins GAP insensitive [33].

**Figure 1:**
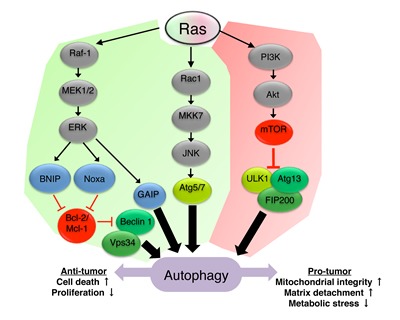
Ras signaling regulates autophagy Ras-mediated upregulation (green) or downregulation (red) of autophagy is depicted in the scheme. Ras can promote autophagy via the Rac1/MKK7/JNK pathway and subsequent upregulation of Atg5/Atg7 [42, 44]. Ras was also shown to induce autophagy through the Raf-1/MEK1/2/ERK pathway which inhibits the binding of Bcl-2/Mcl-1 to beclin 1 leading to the formation of the class III PI3K complex [38, 41], or in a GAIP-mediated manner [40]. Inhibition of autophagy by Ras is mediated by the activation of the class I PI3K/Akt/mTOR1 pathway and subsequent inhibition of the ULK1/Atg13/FIP200 complex [35, 37]. Autophagy induced by Ras can, in turn, affect tumor progression by modulating cell death, cell proliferation, mitochondrial integrity and sensitivity to matrix detachment and metabolic stress.

The crosstalk between Ras and autophagy is well documented and highly complex. Given the known role of Ras as a positive regulator of the class I PI3K/Akt/mTOR1 pathway, it is expected that Ras will act as a negative regulator of autophagy. However, Ras is also involved in the regulation of a vast number of other signaling pathways, therefore its implication in autophagy regulation is in fact multifaceted [30, 34]. Indeed, Ras was shown to have both positive and negative effects on autophagy, depending on the cell type and cellular context (Figure 1).

A classical negative regulation of autophagy by Ras was demonstrated using oncogenic, constitutively active, K-Ras G12V [35]. Expression of K-Ras G12V in NIH3T3 fibroblasts inhibits starvation-induced autophagy mediated by class I PI3K/Akt/mTOR1 pathway. Conversely, dominant negative Ras suppressed the anti-autophagic effect of growth factors. Likewise, in the non-malignant rat intestinal epithelial line IEC-18, H-Ras G12V was found to block autophagy induced by matrix-detachment. This blockage was mediated by RhoA and subsequent activation of calpain, which degrades beclin 1[36]. Another evidence for the autophagy-inhibitory properties of Ras comes from a developmental research in *Drosophila* [37]. During the development of *Drosophila* larvae, growth arrest and cell death occur in the salivary gland, which are associated with increased autophagic cell death. Overexpression of Ras G12V was found to inhibit autophagy as well as salivary gland degeneration. This effect of Ras G12V was partially mediated by the class I PI3K/Akt/mTOR1 pathway.

In opposed to the findings indicating an anti-autophagic role of Ras, *Wu et al* found that oncogenic H-Ras G12V can induce autophagy in NIH3T3 fibroblasts [38]. Activation of autophagy by H-Ras G12V in NIH3T3 cells depends on the Raf-1/MEK1/2/ERK pathway, which upregulates the transcription of BCL2/adenovirus E1B 19 kDa protein-interacting protein 3 (BNIP3). BNIP3 then induces the release of beclin 1 from Bcl-2, thus promoting autophagy. Similarly, BNIP3 was also previously shown to induce autophagy by binding Rheb and inhibiting the mTOR1 complex [39]. Initiation of autophagy through the Ras/Raf-1/MEK/ERK pathway was demonstrated as well by *Pattingre et al* using mutants of H-Ras that differentially activate the Raf-1/MEK/ERK, the PI3K/Akt or the RalGDS pathways [40]. In this work, the expression of H-Ras mutant, which activates only the Raf-1/MEK/ERK pathway (G12V, T35S) in HT-29 cells, was found to activate the autophagic process. Using an inducible system, *Elgendy et al* have also shown that induction of ERK by H-Ras G12V activates autophagy in the non-cancerous HOSE cell line [41]. According to this study, H-Ras G12V enhances the levels of beclin 1 and the BH3-only protein Noxa through the ERK signaling pathway. Noxa displaces Mcl-1 from beclin 1, thus promoting autophagy. H-Ras G12V also promotes autophagy in Rat2 fibroblast, as detected using retroviral expression vector [42]. In these cells, H-Ras G12V activates the Rac1/MKK7/JNK pathway, which in turn, upregulates Atg5 and, subsequently, autophagy. Although this was not tested in this model, active JNK is also known to upregulate autophagy through phosphorylation of Bcl-2 anti-apoptotic proteins, which leads to their release from beclin 1 [43]. In the non-cancerous breast cell line, MCF10A, expression of K-Ras G12V leads to cell transformation along with an increase in Atg5 and Atg7 protein and mRNA levels. This has also resulted in enhancement of autophagy [44]. Interestingly, expression of K-Ras G12V encourages the formation of reactive oxygen species (ROS) and the activation of JNK in MCF10A cells, which mediates the upregulation of Atg5 and Atg7 and subsequently leads to autophagy induction. Similarly, introducing K- or H-Ras G12V into non-tumorigenic iBMK cells was reported to stimulate mTOR1 activation accompanied by higher levels of basal autophagy [45]. Therefore, in these cells, Ras induces autophagy despite mTOR1 activation.

### The role of Ras-induced autophagy

As discussed above, Ras can regulate autophagy through different mechanisms. Autophagy, in turn, can regulate tumorigenicity driven by Ras and dictate cell fate. In MCF10A cells transformed with K-Ras G12V, suppression of Ras-induced autophagy inhibits anchorage independent growth and tumor formation in nude mice, indicating pro-cancer effect of autophagy [44]. In normal MEF cells overexpressing oncogenic H-Ras, autophagy enables cells to escape senescence and promotes cellular tumorigenicity, whereas ASPP2 (Apoptosis-stimulating of p53 protein 2), suppresses this effect by blocking autophagy [46]. In another study, it was shown that autophagy, promoted by oncogenic Ras, supports tumor cells survival by maintaining functional metabolism and mitochondrial integrity [45]. In this case, overexpression of K- or H-Ras G12V initiates autophagy in normal, non-tumorigenic iBMK cells. It was also demonstrated that autophagy-impaired iBMK cells, which overexpressed oncogenic Ras, were more prone to cell death following nutrient depletion, and formed smaller tumors in nude mice. These findings indicates that Ras-induced autophagy facilitates tumorigenesis and is pro-survival for the tumor cells [45]. Interestingly, p62/SQSTM1 impairment had also reduced the viability and tumorigenicity of Ras-expressing iBMK cells [45]. p62/SQSTM1 is a ubiquitin-binding protein, known to mediate the degradation of ubiquitinated proteins and mitophagy [11, 12]. It was found that autophagy-impaired iBMK cells expressing Ras, exhibited some characteristics of defective mitophagy [45]. Hence, autophagy induced by Ras is, apparently, required for functional mitochondrial oxidative metabolism and proper mitochondrial turnover. In corroboration with the results obtained in iBMK cells, it was recently found that autophagy provides a mitochondrial-related metabolic advantage in lung cancer [47]. According to these observations, in K-Ras G12D-driven NSCLC (non-small cell lung cancer) mice model, tumor tissue exhibited high levels of basal autophagy compared to normal tissue. While autophagy-competent tumors progressed to adenocarcinomas, impairment of autophagy reduced tumor grade to benign oncocytomas. Additionally, even when autophagy impaired tumors lacked p53 tumor suppressor protein, they still progressed into oncocytomas. However, these oncocytomas displayed lipid droplets accumulation, presumably due to the presence of dysfunctional mitochondria and insufficient fatty acids oxidation. In contrast, loss of p53 in autophagy-competent tumors only accelerated adenocarcinoma development, and did not result in lipid droplet accumulation [47]. Cells isolated from the autophagy-deficient p53−/− tumors had high basal lipid levels, were more sensitive to starvation and could not form solid tumors in nude mice [47]. Taken together, these data indicate that autophagy promotes NSCLC, especially under metabolic stress, caused by the loss of p53. Another metabolic tumor-promoting role of autophagy in cells expressing oncogenic Ras was reported following matrix detachment [48]. Loss of matrix contact increases autophagy in H-Ras G12V-expressing MCF10A and MEF cells and in cancer cell lines which express mutated K-Ras (Panc-1, HCT-116 and MDA-MB-231). In these cells, autophagy-deficiency limits post-detachment proliferation. The pro-tumorigenic role of autophagy following loss of matrix attachment is mediated by maintenance of glycolytic activity [48]. A similar pro-oncogenic role for autophagy was also suggested in a mutated K-Ras-driven murine model for pancreatic cancer. Using this system, it was demonstrated that chloroquine (an autophagy inhibitor) treatment increases mice survival [10].

Conversely, other studies have shown that autophagy suppresses tumorigenicity driven by mutant Ras, rather than promotes it. In NIH3T3 cells, autophagy induced by oncogenic H-Ras G12V is mediated by BNIP3 and was proposed to act as an anti-proliferative mechanism at short times following Ras overexpression, whereas at longer times autophagy actually promotes cell proliferation [38]. Moreover, overexpression of BNIP in Ras-driven tumor xenografts increases autophagy levels and causes a decrease in tumor weight. In Rat2 fibroblast, H-Ras G12V overexpression induces autophagic cell death that depends on JNK activation and subsequent Atg5 upregulation [42]. In agreement with these findings, H-Ras G12V overexpression also induces autophagic cell death in the non-cancerous HOSE cell line [41]. In this case, H-Ras G12V affects autophagy through Noxa and inhibition of the association between Mcl-1 and beclin 1, as described above. *Yoo et al* had discovered that in the non-malignant rat intestinal epithelial cells, IEC-18, oncogenic H-Ras expression inhibits autophagy and enhances anchorage-independent growth. This pro-tumorigenic effect of H-Ras is reversed by beclin 1-mediated autophagy [36]. Furthermore, ablation of oncogenic K-Ras in human colon cancer cell line, HCT-116, upregulates beclin 1 levels, as well as induces autophagy and inhibits anchorage-independent growth [36].

It was demonstrated that overexpressing oncogenic H-Ras G12V in several human cancer cell lines, which naturally posses wild type forms of Ras (such as U251 and MKN-1, glioma and gastric cancer lines, respectively), induces cytotoxic autophagy [49]. This phenomenon might represent a cellular disposal mechanism set in response to de-regulated oncogene expression. Indeed, Ras mutations are relatively rare in gliomas and gastric cancers [50-53]. Ras-induced autophagic cell death was also suggested to act as a tumor suppressor mechanism in neuroblastoma [54], a tumor which displays high frequency of spontaneous regression [55]. In fact, H-Ras expression was found to correlate with favorable neuroblastoma prognosis [56]. Furthermore, H-Ras expression is also known to correlate with areas of cell degeneration in samples from neuroblastoma patients. Such cell degeneration associated with Ras expression in neuroblastoma specimens resembles autophagic cell death, rather than apoptosis [54]. Accordingly, overexpression of H-Ras (wt or G12V) in neuroblastoma cell lines leads to cell death with autophagic characteristics, and inhibits colony formation [54]. It is, hence, reasonable to conclude that autophagic cell death induced by Ras drives the underlying mechanism of tumor regression in neuroblastoma.

Autophagy was also found to be involved in the degradation of Ras [57]. In malignant peripheral nerve sheath tumor cells, 4-Hydroxy-tamoxifen (OHT) induces caspase-independent cell death accompanied by the degradation of K-Ras. Inhibition of autophagy using 3-MA or siRNA against Atg7 prevents K-Ras degradation and cell death, suggesting that K-Ras is degraded through autophagy following OHT treatment, which subsequently, leads to cell death [57].

### Anti-Ras compounds and autophagy

Currently, there are no available anti-cancer drugs targeting Ras oncoprotein. However, several compounds that were shown to inhibit Ras and suppress tumorigenesis *in vitro* and *in vivo* are under development or in clinical trials [5, 58, 59]. Farnesyltransferase inhibitors (FTIs) are agents designed to block Ras activation by interfering with the addition of farnesylisopernoid residue to the protein, thus inhibiting its anchorage to the cell membrane [5]. The potential of FTIs to modulate autophagy, as well as the role of autophagy following FTIs treatment, was tested by several groups (Table 1). *Pan et al* found that the FTIs manumaycin A, lonafarnib, and FTI-276, which are known to inhibit Ras and Ras-driven tumors [60-62], also induce autophagy in Panc-1 and U2OS cells (human pancreatic cancer and osteosarcma, respectively [63]). Although 3-MA inhibited this FTIs-induced autophagy and led to enhancement of apoptosis, it had no effect on the FTIs-induced inhibition of anchorage-independent growth. Thus, the effect of autophagy induced by manumycin A, lonafarnib and FTI-276 on tumor cell tumorigenicity is yet unknown [63]. Another study had revealed that the combination of FTI-1 and lovastatin induces robust accumulation of LC3-II and autophagosomes in human malignant peripheral nerve sheath tumor and murine hepatoma cell lines [64]. Interestingly, while lovastatin or FTI-1 alone did not affect cell viability and Ras farnesylation, the combination of these two agents reduced farnesylation of Ras, which was accompanied by growth arrest and a non-apoptotic cell death. The profound increase in LC3-II and autophagosomes levels following combined treatment with FTI-1 and lovastatin was found to result from nonproductive autophagy, since neither autophagic flux nor lysosome-autophagosome fusion were detected. Thus, lovastatin and FTI-1 co-treatment might exert its toxic effect as a consequence of aborted/blocked autophagy in the tumor cells tested [64].

**Table 1:** anti-Ras compounds inducing autophagy in cancer cells

Drug tested	Cancer model	Mechanism of anticancer effect	Mechanism of autophagy induction	Role of autophagy in cancer cells	Mode of autophagy inhibition tested
**Manumycin A, lonafarnib and FTI-276 [63]**	Pancreatic cancer and osteosarcoma cell lines	Inhibition of farnesyltransferase; decreases Ras farnesylation and inhibits Ras anchorage to the cell membrane.	Not tested	Uncertain	**Pharmacological: 3-MA**
**FTI-1 [64]**	Malignant peripheral nerve sheath tumor and murine hepatoma cell lines	Inhibition of farnesyltransferase; decreases Ras farnesylation and inhibits Ras anchorage to the cell membrane.	Not tested	Cell destructive	-
**FTS [70, 71]**	Colon adenocarcinoma, cervical cancer and pancreatic cancer cell lines; mouse fibroblasts	Mimicking the farnesyl group of Ras; dislodges Ras from the cell membrane.	mTOR1 inhibition (possibly)	Cell protective	**Pharmacological: 3-MA, Chloroquine** **Genetic: knockout of *ATG5***
**Cysmethynil [72]**	Prostate cancer cell line	Inhibition of Icmt1; decreases Ras methylation and leads to mislocalization of Ras.	mTOR1 inhibition	Cell destructive	**Pharmacological: 3-MA** **Genetic: knockdown of Atg5**
**Cysmethynil [73]**	hepatocellular carcinoma and breast cancer cell lines; mouse fibroblasts	Inhibition of Icmt1; decreases Ras methylation and leads to mislocalization of Ras.	Not tested	Cell destructive	**Pharmacological: 3-MA** **Genetic: knockdown of Atg5 or Atg1/ULK1; knockout of *ATG5***
**Methotrexate [74]**	Osteosarcoma cell lines	Inhibition of Icmt1; decreases Ras methylation and disrupts its proper localization.	Class III PI3K complex activation (dependent upon ULK1/Atg13/FIP200 complex formation)	Cell protective	**Genetic: knockdown of beclin 1 or Atg7**

Other agents have the potential of inhibiting Ras by mimicking its farnesyl group [5]. S*-trans, trans*-farnesylthiosalicylic acid (FTS, also known as Salirasib) is a Ras farnesylcysteine mimetic which acts as a functional Ras antagonist, affecting Ras-membrane interactions, dislodging it from the membrane anchoring domains and facilitating its degradation [65]. FTS was shown to exhibit anti-tumorigenic effects *in vitro* and *in vivo* [66-69]. Treatment with FTS was shown to activate autophagy in wild-type MEF cells and in cancer cell lines harboring a K-Ras mutation (HCT-116, DLD-1, and Panc-1) [70, 71] The effect of FTS on autophagy may depend on mTOR1 inhibition, at least in certain cell types. Knockout of *Atg5* sensitizes MEF cells to FTS-induced growth inhibition and cell death. Moreover, suppression of autophagy with 3-MA or chloroquine in FTS-treated cancer cells enhances FTS-induced growth inhibition, apoptosis and inhibition of anchorage-independent growth. Thus, FTS-induced autophagy seems to play a pro-tumorigenic role. Interestingly, rat fibroblasts transformed with oncogenic H-Ras G12V are more sensitive to the combined treatment with FTS and autophagy inhibitors compared to normal fibroblasts, indicating the specificity of the treatment [70, 71].

In addition to FTI's and farnesyl mimetics, the two less explored Ras modifications have also been considered as targets for anti-Ras inhibitors. Such compounds inhibit Rce1 and Icmt1 enzymes which mediate the cleavage of the terminal AAX sequence of Ras and its subsequent carboxymethylation, respectively [5]. Recent studies had provided evidence for the potential of Rce1 and Icmt1 inhibitors for repressing Ras tumorigenicity, though it is possible that their anti-cancer effect is due to the inhibition of other CAAX-terminating small GTPases (e.g. Rheb, Rac and Ral) [5]. One such inhibitor is cysmethynil, which was found to inhibit cell growth and tumorigenicity in an Icmt1-dependent fashion in cancer cells, accompanied by mislocalization of Ras [5]. As reported by *Wang et al*, cysmethynil inhibits growth of PC3 prostate cancer cells, and is also effective in controlling tumor growth in a xenograft murine model for prostate cancer [72]. Additionally, cysmethynil induces autophagy accompanied by a non-apoptotic cell death in PC3 cells. Cell death probably results from autophagy, as it could be blocked by 3-MA or by knockout of *Atg5*. Cysmethynil was also found to downregulate the class I PI3K/Akt/mTOR1 pathway, which might explain its pro-autophagic effect. The same group has also studied the effect of cysmethynil in hepatocellular carcinoma cell lines and in MDA-MB-231 breast cancer cells [73]. Here, it was found that cysmethynil induces apoptosis, as well as robust autophagy, through specific inhibition of Icmt1. Once again, autophagy induced by cysmethynil presumably contributes to cell death; however, in this setting, it was suggested that excessive autophagy is not the actual mechanism of death, but rather acts a trigger for apoptosis. This notion is attributed to the fact that pharmacologic or genetic suppression of autophagy had also led to the inhibition of apoptosis. Methotrexate, a drug approved for the treatment of high-grade osteosarcoma, is another inhibitor of Icmt1 which was found to decrease Ras methylation by nearly 90%, and also to disrupt its proper localization [5]. *In vitro*, methotrexate was reported to induce autophagosome formation accompanied by the upregulation of HMGB1 mRNA and protein levels in osteosarcoma cell lines [74, 75]. HMGB1 functions as a DNA chaperone protein, and was linked to multiple cancers in humans [74]. Upregulated HMGB1 was suggested to compete with Bcl-2 for the binding to beclin 1 thus enhancing the formation of the class III PI3K complex [74, 75]. The interaction of HMGB1 with beclin 1 apparently depends upon the ULK1/Atg13/FIP200 complex formation, as it was inhibited by knocking down ULK1 or FIP200. It was demonstrated that methotrexate treatment combined with genetic inhibition of autophagy had lead to apoptosis, and reversed the drug resistance in osteosarcoma cells [74]. Likewise, the knockdown of HMGB1 increased sensitivity to methotrexate *in vivo* [74]. Hence, it is reasonable to suspect that methotrexate induces tumor-promoting autophagy in osteosarcoma cells by upregulating HMGB1.

## CONCLUDING REMARKS

The fact that oncogenic Ras can have opposite effects on autophagy induction emphasizes its dual role in the regulation of this process. A possible explanation for this ambivalence is that oncogenic Ras activates both pro- (i.e. JNK and ERK) and anti-autophagic (i.e. class I PI3K/Akt/mTOR1) signaling pathways, depending on cellular context (Figure 1).

Another conflict, which arises from the findings, is that Ras-induced autophagy has both pro- and anti-tumorigenic roles. However, one might speculate that the stress inflicted on the cells will determine the role of autophagy induced by Ras. As described above, nutrient depletion and metabolic stress, mitochondrial dysfunction, matrix detachment and senescence might represent stress conditions in which autophagy supports cell viability and, thus, acts as a tumor promoting mechanism [4, 44, 46-48]. Indeed, under such conditions autophagy is known to be cell protective [1, 76-78]. In contrast, under other autophagy activating conditions, it can have a tumor-suppressive outcome [36, 38, 41, 42, 49, 54]. Another explanation for the apparently opposing roles of Ras-induced autophagy might relate to the progression of Ras-driven tumorigenicity. It is plausible that unregulated Ras activity imposes different demands on the cells at early/late stages of malignant transformation (in terms of energetic and biosynthetic requirements, for example). It was suggested, that at early stages of oncogenic transformation, Ras-induced autophagy acts as a cell death mechanism, targeted to the elimination of tumor cells, thus preventing tumor progression [79]. However, cells that overcome this bottleneck effect, caused by autophagic cell death, become more aggressive and more resistant to the toxic effect of autophagy. In such cells, autophagy may now serve as a cell-protective mechanism, especially under stress conditions [79]. Furthermore, at early stages of Ras oncogenesis, autophagy may contribute to protein quality control and assist in degradation of overexpressed/altered Ras proteins [57], whereas at later stages this function may become inefficient. The notion that autophagy is required to sustain tumor growth at late stages of tumor development is further supported by the fact that several oncogenic Ras-expressing cancer cell lines (e.g. HCT-116, Panc-1, MDA-MB-231, DLD-1, SW480) exhibit high levels of basal autophagy, which seems to be protective [10, 48, 80] Indeed, it was suggested by *Mancias and Kimmelman* that Ras-transformed cells may be addicted to autophagy in order to evade metabolic stress and cell death [4]. In light of this, the consequence of Ras-induced autophagy may differ, depending on the level of tumorigenicity.

It is evident that Ras inhibitors promote autophagy in cancer and non-cancer cells, though the role of autophagy induced by these agents is yet inconclusive (Table 1) [63, 64, 70-74]. Likewise, it is not clear whether autophagy triggered by Ras inhibitors actually originates from the inhibition of Ras or other small GTPases containing a farnesyl group. Indeed, the aforementioned inhibitors: FTS, cysmethynil and FTI-1, were shown to inhibit also the farnesylated small GTPase Rheb, which negatively regulates autophagy [64, 72, 81, 82].

In conclusion, Ras and autophagy may have dual effect on cancer progression depending on the cellular context. This should be carefully considered when designing novel anti-cancer therapeutics based on Ras and autophagy modulation.
